# FDHE-IW: A Fast Approach for Detecting High-Order Epistasis in Genome-Wide Case-Control Studies

**DOI:** 10.3390/genes9090435

**Published:** 2018-08-29

**Authors:** Shouheng Tuo

**Affiliations:** School of Computer Science & Technology, Xi’an University of Posts & Telecommunications, Xi’an 710121, China; tuo_sh@126.com

**Keywords:** Single-nucleotide polymorphism, high-order epistasis, interaction weight

## Abstract

Detecting high-order epistasis in genome-wide association studies (GWASs) is of importance when characterizing complex human diseases. However, the enormous numbers of possible single-nucleotide polymorphism (SNP) combinations and the diversity among diseases presents a significant computational challenge. Herein, a fast method for detecting high-order epistasis based on an interaction weight (FDHE-IW) method is evaluated in the detection of SNP combinations associated with disease. First, the symmetrical uncertainty (*SU*) value for each SNP is calculated. Then, the top-k SNPs are isolated as guiders to identify *2-way* SNP combinations with significant interaction weight values. Next, a forward search is employed to detect high-order SNP combinations with significant interaction weight values as candidates. Finally, the findings were statistically evaluated using a *G*-test to isolate true positives. The developed algorithm was used to evaluate 12 simulated datasets and an age-related macular degeneration (AMD) dataset and was shown to perform robustly in the detection of some high-order disease-causing models.

## 1. Introduction

In recent years, genome-wide association studies (GWASs) have played an important role in identifying single-nucleotide polymorphisms (SNPs) associated with complex human diseases. This approach is non-candidate-driven, and investigates the entire genome, thus offering a more comprehensive method when compared to gene-specific candidate-driven studies [1]. Genome-wide association studies was first employed by Klein et al. to investigate patients with age-related macular degeneration (AMD), and they identified two SNPs (rs380390 and rs10272438) with significant AMD associations [2]. Since its conception, 1800 diseases and traits, and thousands of associated SNPs have been identified, with the main focus being on individual SNPs that are isolated based on their contribution to disease status [3]. However, more recently, it has become widely accepted that multiple SNPs may contribute to a given pathogenicity via epistasis. Epistasis is defined as the effect of one gene (allele) on a phenotype that is modified by another gene (allele) or several other genes (alleles) [4], and includes additive/synergistic epistasis, dominant epistasis, recessive epistasis, functional epistasis, and sign epistasis [5].

Nevertheless, detecting and identifying the disease-causing SNP combinations on a genome-wide scale is met with several challenges. First, there is an enormous computational burden that is associated with the examination of SNP combinations due to multiple testing. Second, it is challenging to develop a method that is able to reliably identify disease-causing SNP combinations from those that are not given the diversity that exists among disease models [6], especially when there is insufficient sample data.

To tackle these challenges, some algorithms were developed to detect synergistic SNP combinations associated with complex diseases. The majority of these methods can be classified into three categories: exhaustive methods [7,8,9,10,11], filtering methods (SNPHarvester) [12,13], or artificial intelligence (including swarm intelligence and heuristic search methods) [14,15,16,17,18,19,20,21,22].

In the exhaustive method, all SNP combinations from the original data set are verified, which ensures that disease-causing SNP combinations are seldomly missed, but it bears an enormous computation burden. To speed up the calculations, some lightweight scoring methods are employed. However, they usually prefer a small part of a disease model. The classical exhaustive methods include Boolean operation-based screening and testing (BOOST) [7], graphical processing unit (GPU)-implementation of BOOST (GBOOST) [8], GPU-based tools for parallel permutation tests in GWASs (PBOOST) [9], and epistasis analysis based on multi-objective optimization (ESMO) [10]. Boolean operation-based screening and testing utilizes a Boolean operation to speed up the examination of pairwise SNP interactions using an exhaustive search approach. GBOOST and PBOOST further accelerate detection by employing GPUs. Epistasis multi-objective optimization utilizes exhaustive methods to evaluate all SNP combinations using mutual entropy and a Bayesian network. It is not feasible for high-order epistasis detection, due to the enormous computational burden. 

The filtering method applies data-driven and biological knowledge filters. The data-driven filter reduces SNP numbers by calculating the correlation between individual SNPs and disease status. Single-nucleotide polymorphisms below a given threshold are filtered out, and some disease-causing SNPs with a very low effect may potentially also be removed. The biological knowledge filter groups genes in the light of their biological functions, and detects the epistatic interactions within a group, but introduces a bias towards well-known genes, and against less well-characterized genes [3,12,13].

Artificial intelligent algorithms, such as bayesian epistasis association mapping (BEAM) [14], Ant colony optimization based epistatic interaction (AntEpiSeeker) [15], Cuckoo search epitasis (CSE) [16], multi-objective ant colony optimization epistasis detection (MACOED) [17], fast harmony search algorithm based SNP epistasis detection (FHSA-SED) [18], niche harmony search algorithm based high-order SNP combination detection (NHSA-DHSC) [19], Co-Information basedN-Order epistasis detector and visualizer (CINOEDV) [20], and high-order interaction seeker(HiSeeker) [22] have attracted attention when detecting high-order epistatic interactions, due to a reduced computational burden, which is due to not all SNP combinations being examined. However, these algorithms are often sensitive to parameters, and easily trapped in local searches [23,24].

**Figure 1:**
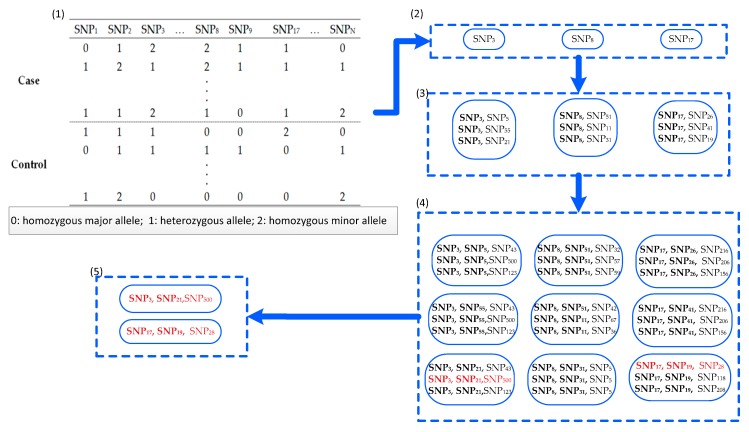
An example of the fast approach for detecting high-order epistasis with interaction weight (FDHE-IW) approach for detecting *3-way* single nucleotide polymorphism (SNP) combinations associated with a given phenotype. (1) Using the dataset with N SNPs, the *SU* value of each SNP was calculated. (2) The top three SNPs with the largest *SU* values were selected from the N SNPs, and then the three SNPs were employed as seeds to calculate the *2-way* interaction weights (*IW*) with the other SNPs. (3) The top nine *2-way* SNP-combinations were selected from the 2-way SNP combinations that pair with the three parent SNPs from (2), based on *IW* values. (4) The top 9 × 3 = 27 3-way SNP-combinations that are formed from a parent SNP-combination in (3) were selected based on *IW*. (5) The *G*-test statistical method was employed to test the 27 *3-way* SNP combinations, and two *3-way* SNP combinations were verified using the G-test.

Recently, information theory has focused on selection from high-dimensional data sets [25,26,27]. In this study, an approach termed a fast approach for detecting high-order epistasis with interaction weight (FDHE-IW) was developed. In this approach, *k-way* epistasis interactions are detected, with the detection process divided into two stages: searching and testing. During the searching stage, an improved method is utilized to calculate the symmetrical uncertainty (*SU*) of each SNP locus with a given phenotype. Next, several SNPs with high *SU* values are chosen as guiders to isolate *2-way*, *3-way*, and *k-way* SNP combinations that are significantly associated with disease status. These SNP combinations are recorded as candidate solutions. During the testing stage, the *G*-test statistical method was employed to test the association significance of the candidate solutions. An example schematic for detecting *3-way* SNP combinations can be seen in Figure 1.

## 2. Materials and Methods

Sets of SNP variables are defined by $X=\{X_{1},X_{2},\cdots ,X_{N}\}$ with N SNP loci, where *X_i_* is the genotype variable of the *i*th SNP locus with values of $\left\{x_{i}^{1},x_{i}^{2},\cdots ,x_{i}^{n}\right\}$, accounting for the homozygous major allele (0), the heterozygous allele (1) and the homozygous minor allele (2). C denotes the phenotype variable with values of $\left\{c_{1},c_{2},\ldots ,c_{J}\right\}$ (J=2 for a given disease phenotype). For a *k-way* SNP combination $\{X_{a_{1}},X_{a_{2}},\cdots ,X_{a_{k}}\}\left(1\leq a_{i}\leq N,1\leq i\leq k\right)$ (such as a *3-way* SNP combination $\left\{X_{3},X_{8},X_{150}\right\}$), *I* denotes the number of genotype combinations (*I* = 3***^k^*** for *k-way* SNP combination), *J* is the number of phenotype states (C), with *J* = 2 for a given disease phenotype (it equals 2 for a case-control dataset. 1 denotes case label, 0 is the control label). The number of samples in a dataset is defined as *n_i_*, with the SNP loci taking the value of the *i*th genotype combination (1 ≤ *i* ≤ *I*), and *n_ij_* representing the number of samples of the *i*th genotype combination being associated with the phenotype state (*c*_j_).

### 2.1. Definitions

“Information entropy” has been defined as the average amount of information that is produced by a stochastic source of data that can be used to measure the data distribution diversity, and to measure the uncertainty of random variables [28]. To introduce our method well, the terms were defined:

*Entropy*: Let $p(x_{i})$ be the probability of the *i*th genotype of a SNP variable (*X*), with the entropy of *X* being expressed as:

(1) $$H(X)=-\sum_{i=1}^{n}p(x_{i})\log p(x_{i})$$

*Joint entropy*: Let $X_{1},X_{2},\cdots ,X_{k}$ be *k* SNP variables, with $\left(x_{i}{}_{{}_{1}},x_{i}{}_{{}_{2}},\cdots ,x_{i}{}_{{}_{k}}\right)$ being a particular genotype value for these SNP loci, and $p(x_{i}{}_{{}_{1}},x_{i}{}_{{}_{2}},\cdots ,x_{i}{}_{{}_{k}})$ being the probability of the genotype occurring together. The joint entropy (JE) of multiple variables is defined as:

(2) $$H(X_{1},X_{2},\cdots ,X_{k})=-\sum_{i_{1},i_{2},\ldots ,i_{k}}^{I}p(x_{i_{1}},x_{i_{2}},\cdots ,x_{i_{k}})\log p(x_{i_{1}},x_{i_{2}},\cdots ,x_{i_{k}})$$

The JE is a measure of the uncertainty that is associated with a set of variables and can be used to measure the genotype distribution of a *k-way* SNP combination $\left(X_{1},X_{2},\cdots ,X_{k}\right)$; however, it cannot be used in assessing genotype–phenotype correlations. Recently, mutual information has attracted extensive attention for identifying the association.

*Mutual information*: To measure the mutual dependence between a SNP locus (*X*) and phenotype (*C*), the mutual information was defined as follows:

(3) $$I(X;C)=\sum_{i=1\;}^{I}\sum_{j=1}^{J}p(x_{i},c_{j})\log\frac{p(x_{i},c_{j})}{p(x_{i})p(c_{j})}=H(X)+H(Y)-H(X,Y)$$

where p(x) denotes the marginal probability distribution function of *X*.

*Joint mutual information*: The joint mutual information between *k* variables (*X*_1_, *X*_2_, *X_k_*) and the phenotype (*C*) was defined as follows:

(4) $$I([X_{1},\cdots ,X_{k}];C)=H(X_{1},\cdots ,X_{k})+H(C)-H(X_{1},\cdots ,X_{k},C)\;$$

The mutual information can also be regarded as a measure of the interaction strength between two SNPs (*X* and *C*), or between a SNP (*X*) and the disease status (*C*).

*Interaction gain*: Mutual information can also be called an interaction gain (IG) between *X* and *Y*. Based on a previous proposed interaction gain with three variables *X*, *Y*, and *C* [29], the following was derived:

(5) IG(X;Y;C)=I([X,Y];C)−I(X;C)−I(Y;C) 

In GWASs, *X* and *Y* can be regarded as SNP loci, and *C* can be either a SNP variable or phenotype variable. In *IG(X; Y; C)*, a *2-way* interaction between SNP *X* and SNP *Y* for phenotype *C* can be depicted, or a *3-way* interaction between *X, Y*, and C can be indicated. If *X*, *Y*, and *C* are a feature set, such as in $X=\{x_{1},x_{2}\},Y=\{x_{3}\},C=\{y\}$, IG(X;Y;C) can be regarded as an IG between *X* and *Y*.

In Equation (5), if IG(X;Y;C)>0 (I(X,Y;C)>I(X;C)+I(Y;C)) then *X* and *Y* together yield a more synergistic effect on phenotype than would be expected from their sum individually. Thus, *X* and *Y* interacting with each other (interaction effect), and the presence of *Y* will increase the ability of predicting the phenotype (*C*). Conversely, IG(X;Y;C)<0 (I(X,Y;C)<I(X;C)+I(Y;C)) indicates a redundancy between *X* and *Y*.

In GWAS, measuring the interaction between genotype variables (*X*, *Y*) and phenotype *C* is very important but difficult. In this study, a new interaction weight (*IW*) factor was employed.

*Interaction weight factor*: The interaction weight factor (*IWF*) between *X* and *Y* has been previously [30] defined as:

(6) $$IWF(X,Y)=1+\frac{IG(X;Y;C)\;}{H(X)+H(Y)}$$

*IWF(X, Y)* has the following properties:

(1) 0 ≤ *IWF(X, Y)* ≤ 2
(2) 1 ≤ *IWF(X, Y)* ≤ 2 if *X* interacts with *Y*.
(3) 0 ≤ *IWF(X, Y)* ≤ 1 if *X* is redundant to *Y*.

Evaluating the association of all SNP combinations with specific phenotypes may be time-consuming, and the computational evaluation of high-order SNP combinations is generally difficult to perform. To speed up the process of detecting epistasis from high-dimensional data sets, the present study employed Symmetrical uncertainty in identifying search seeds.

*Symmetrical uncertainty (SU*): Mutual information has been widely adopted for data mining from high-dimensional data. However, it tends to favor features with more values while ignoring interactive features [31]. In this study, SU was utilized to compensate for the bias of mutual information toward features with more values, as previously described [27,30], and this was defined as Equation (7):

(7) $$SU(X;C)=\frac{2I(X;C)}{H(X)+H(C)}$$

In Equation (7), I(X;C) I(X;C) is used to measure the mutual dependence between the SNP locus (*X*) and the disease status (*C*). *H*(*X*) and *H*(*C*) measure the diversity of the SNP genotype distribution and the phenotype, respectively. Nevertheless, *SU* cannot effectively measure low associations. To enhance the ability to detect lower SNP and phenotype associations, the *SU* equation was modified as follows:

(8) $$SU(X;C)=\frac{2I(X;C)\;}{H(X,C)}$$

In Equation (8), *H*(*X*) + *H*(*C*) is replaced with *H*(*X*, *C*) because we have found that the joint distribution of variables X* and *C* (let X* be the disease locus) usually have a smaller degree of dispersion than those of the other variables, namely, *X* and *C*. This improved Equation (8) thus enables the identification of some susceptibility loci with low marginal effects. This proposed algorithm is further described in Algorithm 1.


**Algorithm 1: FDHE-IW**
**Inputs**: **D** (*s*_1_, *s_2_*, *…*, *s_N_*_,_
*C*)—the given data set with *N* + 1 columns; *s_i_* denotes the values of the *i*th SNP locus for all samples. *T*—the candidate size; θ—the threshold of the *G*-test *p*-value; *k*—the number of SNPs in a *k-way* SNP combination; and *K*—the number to find the SNP combinations based on a seed SNP. **Outputs**: SNP combinations (SC)—the *k-way* SNP combinations that are associated with disease status.
(1)**Initialize**: $S^{0}=\left\{\;s_{1},\;s_{2},\ldots ,s_{N}\right\},\;SC=\emptyset ,k=0,F=S^{0}$(2)**Calculate** the SU for each SNP. **For *i* = 1 to *N* do**  Calculate $SU\left(s_{i},C\right),s_{i}\in S^{0}$  $W\left(s_{i}\right)\leftarrow 1$ **End For**

**(3)** 
**Search a *k-way* SNP combination based on the interaction weight.**
**(3.1)** **Select a SNP locus with a maximum**SU×W**value**. $s_{a}\leftarrow \underset{s_{i}\in F}{\arg\max}\;SU(s_{i},C)\times W(s_{i}),i=1,2,\ldots ,N$**(3.2)** 
**Search SNP combination based on interaction weight**

***m* = 1**

**While *m* < *K* || // to find *K k-way* SNP combinations based on** $s_{a}$
   S ←∅    $\begin{array}{l} S\leftarrow S\cup \{s_{a}\}\\ F\leftarrow F\backslash \{s_{a}^{}\}\\ W(s_{a})\leftarrow 0 \end{array}$    **While** |S| **< *k //*** |S| is the SNPs number in *S*.     **For *i* = 1 to |*F*| do** // |*F*| denotes the SNPs number in *F*.        $IW\;\left(s_{i}\right)\leftarrow \;IWF\left(s_{i},s_{a};\;C\right),\;s_{i}\in F$ // Calculate interaction weight between *s_i_* and *F_a_*:        $W\left(s_{i}\right)\leftarrow W\left(s_{i}\right)\times IW\left(s_{i}\right),s_{i}\in F$ // Update the **weight coefficient**.        *//* calculate relevance between $s_{i}$ and phenotype (*C*)        $R\left(s_{i},C\right)\;\leftarrow \;W\left(s_{i}\right)\times \left(1+SU(s_{i},\;C\right)),\;I=1,2,\ldots ,|S|$     ***End For***$s_{a}\leftarrow \underset{s\in F}{\arg\max R(s,C)}$ // Select out the SNP $s_{a}$ that has maximum relevance with *C* in *F*.     $S\leftarrow S\cup \{s_{a})$     $F\leftarrow F\backslash s_{a}$ // remove SNP $s_{a}$ from *F.*   ***End While***   SC←SC∪{S} // Store the found SNP combination *S* into *SC* as a candidate solution.   ***m = m + 1***  **End While****(4)** **If** the size of *SC* is less than *T*
**go to step (3) to find new *k-way* SNP combination that are associated with disease status.**

**EndIf**
**(5)** 
**Statistical test**
Perform *G*-test statistic for each SNP combination in *SC*.Output the *k-way* SNP combinations with a *p*-value < θ


The FDHE-IW algorithm first calculates the *SU* value for each SNP (Step (2)). Then the SNP $s_{a}$ with a maximum *SU* value is chosen (see step (3.1)) to find *K k-way* SNP combinations that are associated with the status of the diseases. In step (3.2) of the FDHE-IW algorithm, the *IW* and weight coefficient (*W*) for each SNP in *F* are updated iteratively, and a new SNP having a maximum relevance with the phenotype is combined with SNP $s_{a}$.

*Time Complexity*: In the FDHE-IW algorithm, the time complexity is defined as *O*(*N* + N*(*k*!)*K). Generally, the values of *k* and *K* are very small, and the value of (*k*!)*K is much less than *N*. Therefore, the time complexity of FDHE-IW is less than *O*(*N*^2^), which is a feasible computation amount for current computers to detect high-order SNP epistasis from a data set with thousands to millions of SNPs.

*G-Test*: A *G*-test is a maximum likelihood statistical significance test [32]. Compared to a Chi-square test, the *G*-test will lead to the same test results for samples of a rational size. However, for some cell cases, it is always better than the Chi-squared test [33].

In this study, an improved *G*-test method [19] was employed to verify the association between genotype and phenotype. For the *k-way* SNP combination model, the formula for calculating the *G* value is as follows:

$$G=2\sum_{i=1\;}^{I}\sum_{j=1}^{J}O_{ij}\cdot P_{ij}$$

$$P_{ij\;}=\{\begin{array}{c} \ln\frac{O_{ij}}{E_{ij}},\sum_{j=1}^{J}O_{ij}>\xi \\ 0,otherwise \end{array}$$

The degree of freedom *d* (*d* = (*I* − 1)(*J* − 1)) is modified correspondingly, as follows:

$$\begin{array}{l} d=(I-1)(J-1)\\ for\;i=1\to I\\ \begin{array}{cc} \; & if\sum_{j=1}^{J}O_{ij}<\xi \end{array}\\ \begin{array}{ccc} & & d=d-1 \end{array}\\ \begin{array}{cc} & endif \end{array}\\ endfor \end{array}$$

where, $O_{ij}$ and $G=2\sum_{i=1}^{I}\sum_{j=1}^{J}Q_{ij}\cdot P_{ij}$ are the observed numbers and the expected number of genotypes, respectively, when the phenotype takes the state *y_j_*, and the genotype takes the *i*th *k*-combination*.* ln denotes the natural logarithm function. The observed number $G=2\sum_{i=1}^{I}\sum_{j=1}^{J}Q_{ij}\cdot P_{ij}$ is obtained from the dataset by using a simple counting statistical method. The expected genotype frequency number (*E_ij_*) is obtained according to Hardy–Weinberg principles [34]. ξ is a small integer that is less than or equal to 5.

### 2.2. Performance Evaluation and Simulation Data Sets

#### 2.2.1. Performance Evaluation

To evaluate the performance of the proposed algorithm, equations for the power Equation (10), the F-measure Equation (11), the recall Equation (12), and the precision Equation (13) were utilized.

(9) $$Power=\frac{\#TP\;}{\#S}$$

(10) $$F-measure=\frac{2}{1/recall+1/precision\;}$$

(11) $$recall=\frac{\#TP\;}{\#TP+\#FN}$$

(12) $$precision=\frac{\#TP\;}{\#TP+\#FP}$$

Power is a measure of the capability to detect disease-causing models in all datasets, where *#S* is the number of disease-causing models from all *#T* datasets (there are 100 data matrices for each disease model).

True positives (TPs) are defined as the discovery of a *k-way* SNP combination that is associated with disease status, and FNs (false negatives) are defined as a non-discovery of a SNP combination that is associated with disease. TNs (true negatives) indicate no discovery, and FPs (false positives) are defined as a *k-way* SNP combination that is falsely associated with a disease status [31].

In this experiment, *recall*, *precision* and *F-measure* were used to evaluate the statistical precision of this hypothesis testing method (*G*-test in our method) for finding disease models in the screening stage. *#TP* is equal to the number of disease-causing SNP combinations that have passed the threshold *p*-value, while *#FN* is the number of the disease-causing SNP combinations that failed to pass the threshold. *#FP* is the number of non-disease-causing combinations that passed the threshold, while *#TN* equals the number of non-disease-causing combinations that failed to pass the threshold.

#### 2.2.2. Simulation Data Sets and Case Study

Twelve disease loci with marginal effects (DME)-simulated disease models (multiplicative models: DME-1–DME-4, threshold models: DME-5–DME-8, and concrete models: DME-9–DME-12) (see Tables S1 and S2 in Supplementary File 2) that are well characterized were utilized [17], with 100 simulated data sets being generated for each DME disease model using GAMETES_2.0 [35]. One of the generated data sets contained 100 SNPs, with 800 controls and 800 cases; while another contained 1000 SNPs, with 2000 cases and 2000 controls. The experimental results of our algorithm were then compared with BEAM [14], BOOST [7], MACOED [17], and SNPHarvester [12] results. BEAM is a classical heuristic search algorithm for detecting epistasis interactions. It applies a Bayesian partitioning model to disease-associated markers and their interactions, and uses a Markov chain Monte Carlo (MCMC) to compute the posterior probability that each marker set is associated with a given disease [14]. Boost detects the SNP epistasis interaction very rapidly using a filter and an exhaustive method. SNPHarvester is a classical and effective filtering-based approach for detecting epistatic interactions in genome-wide association studies. MACOED is a new intelligent search algorithm for detecting high-order epistatic interactions that reduces the number of SNP combinations that are examined in association with a phenotype.

To further validate the algorithm, real AMD data containing 103,611 genotypes SNPs for 50 controls and 96 cases [14] were utilized. To balance the case samples and controls, we enlarged the control sample size to 96 using a bootstrap method [36], which can significantly increase the statistical power, and imputed missing data using the k-nearest-neighbor method [37].

#### 2.2.3. Paremeters Setting

In the DME simulation experiments, the threshold θ of the *G*-test *p*-value was set equal to $\frac{0.01\times MAF}{C_{N}^{k}}$, with minor allele frequency (*MAF)*. The candidate size set to *T* = 2*k for simulation data sets and *T* = 200 for AMD. *K* = *k* for simulation data sets and *K* = 5 for AMD data. All experiments were performed using a Windows 10 operation system with Intel(R) Core(TM) i7-4790 CPU@3.6GHz and 8 GB memory, and all program codes were written in MATLAB R2015b (MathWorks, Natick, MA, USA). The source code is in Supplementary File FDHE-IW.rar.

## 3. Results

### 3.1. Simulated Models

**Figure 2:**
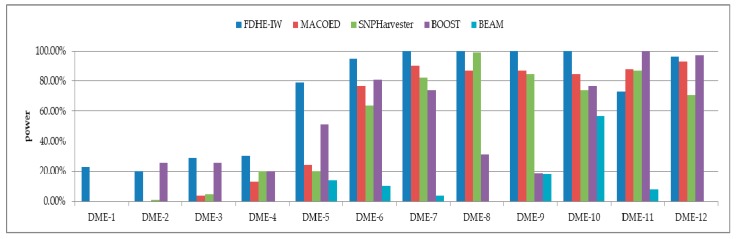
Detection powers of the five evaluated algorithms (100 SNPs, 1600 sample size).DME: Disease loci with marginal effects.

**Figure 3:**
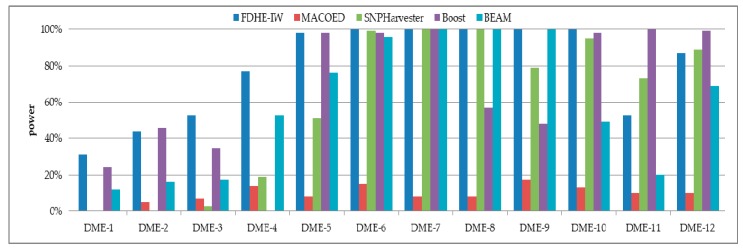
Detection powers of the five evaluated algorithms (1000 SNPs, 4000 sample size).

The detection power of FDHE-IW was first investigated by comparing it with four state-of-the-art algorithms (BEAM, SNPHarvester, MACOED, and BOOST) using a DME dataset with 100 SNPs (Figure 2) and a data set with 1000 SNPs (Figure 3). The results in Figure 2 and Figure 3 show that FDHE-IW is superior to the other four algorithms when analyzing DME-1 and DME-3–DME-10, and is comparable with BOOST for DME-2 and DME-12.

The recall, precision and F-measure were also determined when using FDHE-IW for all 12 DME models (Table 1), with the five comparative algorithms also evaluated using a subset of DME models (Table 2 and Table 3). These results indicated that the performance of FDHE-IW when using the dataset with 1600 samples was inferior when compared to the dataset with 4000 samples in recall, precision, and F-measure.

The results in Table 2 and Table 3 showed that the performance of FDHE-IW was superior to BEAM, SNPHarvester, and BOOST in recall, precision, and F-measure. However, it was found to be inferior to MACOED for most of the DME models. This is because some disease-causing SNP combinations identified by FDHE-IW in the searching stage were then rejected in the testing stage, but in MACOED, only SNP combinations that were significantly associated with disease status were identified. For many disease-causing SNP combinations with a low *MAF* and low hereditary, MACOED fails to identify them in the screening stage. Therefore, the MACOED has higher precision than FDHE-IW in the testing stage, but the detection power of MACOED is much lower than that of FDHE-IW (Figure 2). As can be seen in Table 2 and Table 3, FDHE-IW has a lower runtime than MACOED, BEAM, and SNPHarvester, but it has a longer runtime than Boost.

### 3.2. Experimental Results Using an AMD Dataset

**Figure 4:**
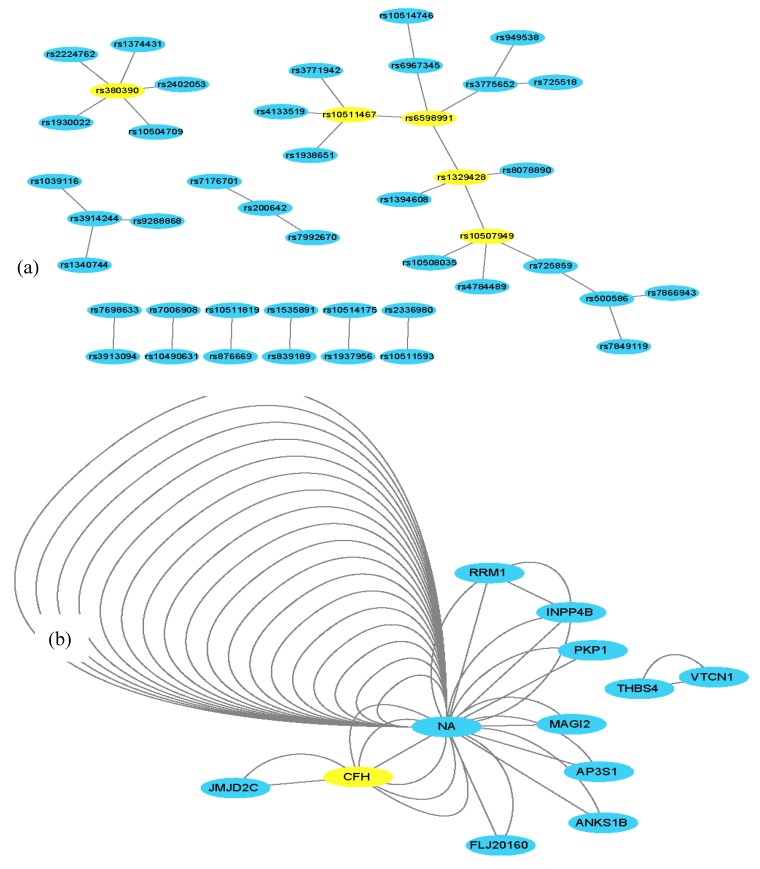
*2-way* SNP and a representative gene network. (**a**) There are 35 edges and 45 nodes in Figure 4a, where each node denotes a SNP locus. An edge represents a *2-way* SNP combination that has a strong association with the phenotype. The yellow SNPs (nodes) have been reported to be associated with age-related macular degeneration (AMD). (**b**) In Figure 4b, the nodes and edges are mapped from nodes and edges from Figure 4a, in which a node denotes a gene, and each edge represents a *2-way* SNP combination that is mapped to two genes. NA denotes non-gene coding regions; there are multiple NA–NA edges because multiple SNP–SNP pairs were mapped to non-gene-coding regions. The greater the number of edges between two gene nodes, the more the SNP combination maps into the two genes. The yellow genes (nodes) are believed to be associated with AMD. In node NA, there are many edges, which means there are multiple SNP combinations in the non-coding region.

To further validate the FDHE-IW algorithm, a real AMD data set was evaluated. It took 5 h to find 200 *2-way* candidate solutions, 15 h to find 200 *3-way* candidate solutions, and 25 h to find 8 *4-way* candidate solutions. The algorithm identified 45 *2-way* SNP combinations (*p*-value < 10^−11^), 18 *3-way* SNP-combinations (*p*-value < 1 × 10^−15^; Table 4) and 2 *4-way* SNP-combinations (*p*-value = 0; Table 5) that were associated with AMD out of the 103,611 examined SNPs (Supplementary File 1, sheets 1-way–*4-way*). The obtained findings were further examined using Cytoscape (http://www.cytoscape.org/) [38], and a *2-way* SNP interaction network and a *2-way* gene interaction network were constructed (Figure 4), where the genes were mapped based on the SNP interaction networks.

Within the SNP network (Figure 4), two widely reported SNPs (rs380390, rs1329428) that are located in an intron within the *CFH* gene were also identified. The *CFH* gene has been commonly association with AMD [14,18,19]. Furthermore, the constructed gene network showed that many of the SNPs are mapped to non-gene coding regions, thus denoted NA. Within the interaction network, NA and *CFH* had six connections due to six SNP pairs within the SNP network being mapped to a *CFH*–NA gene-pair. CFH was also found to have a novel connection with the *JMJD2C* gene, a histone lysine demethylase. *JMJD2C* has been reported to play a crucial role in the progression of breast cancer, prostate carcinomas, osteosarcoma, and blood neoplasms, thus indicating that *JMJD2C* represents a promising anti-cancer target [39,40,41]. These findings also suggest that *JMJD2C* may have an important role in AMD.

Eight *3-way* gene combinations containing the *CFH* gene, and four *3-way* gene combinations containing the *JMJD2C* gene were identified. Only one *3-way* combination did not involve the *CFH* and *JMJD2C* genes, suggesting that these are important to AMD.

The two *4-way* SNP combinations in Table 5 shows little uncertainty on whether every SNP contributes to the phenotype. Therefore, the AUCs (areas under the curve) of each SNP involved in a potential SNP combination (1-way, *2-way*, and *3-way*) were computed and then compared to the AUC relative to the *4-way* SNP combinations (see Figures S1 and S2 in Supplementary File 3). The AUC of the *4-way* SNP combination was larger than those of other sub SNP combinations. Hence, each SNP in the SNP combinations contribute to the development of AMD.

Recent investigations have utilized AMD data sets, which include algorithm IOBLPSO [42], epiACO [43], BEAM [14], epi forest [44], DCHE [45], FHSA-SED [18] and NHSA-DHSC [19]. Table 6 summarizes the results of these seven studies. Two SNPs (rs380390, rs1329428) and the *CFH* gene have been reported by all the seven algorithms. However, other SNPs and SNP combinations that are associated with AMD were detected by different algorithms. The proposed FDHE-IW also detected novel SNPs and genes, where rs10511467 (in NA) and rs3776652 (in the *JMJD2C* gene) were also reported by FHSA-SED and NHSA-DHSC. SNPs rs6598991 and rs10507949, both in NA, have not been reported to date.

## 4. Discussion

In this study, a fast-search method based on the interaction weight was proposed to detect *k-way* SNP epistasis that is associated with disease. When utilizing simulation data sets, the method developed herein was shown to be more powerful than other comparable algorithms in detecting the disease-causing SNP combinations at the searching stage. However, in the testing stage, the balance between type I and type II errors is very difficult to manage, due to the *p*-value threshold for distinguishing between true and false disease-causing combinations being very different for different disease models. Therefore, multiple statistical tests, such as the Chi-square test, *t*-test, and *G*-test, were evaluated, with the *G*-test being shown to be the most robust. When utilizing the newly developed algorithm to evaluate an AMD dataset, almost all of the well-known disease-causing SNP loci associated with AMD and some new SNP combinations were identified. However, the method developed herein identified many SNPs that are in non-coding genomic regions, which will require further examination in future studies. The detection of disease-associated SNPs in high-order disease models is a very difficult problem.

## 5. Conclusions 

### 5.1. Advantage

The FDHE-IW algorithm does not need to evaluate all *k-way* SNP combinations to detect *k-way* disease-causing SNP combinations. It is capable of detecting some higher-order epistasis on a whole genome scale with a time complexity that is much less than *O*(*N*^2^).

### 5.2. Limitations

The proposed algorithm can effectively detection nested epistasis [35], suggesting that one or more of the interacting loci are the major contributors to disease, and that at least one proper subset of the loci also interacts epistatically. However, for some disease-causing SNP interaction combinations with pure, strict epistasis, the detection power of FDHE-IW is still unsatisfactory.

### 5.3. Future Work

At present there is still no fast or effective approach for detecting various disease-causing models with multi-loci in GWAS, due to the enormous computational burden. Therefore, detecting high-order disease models has room to be explored using high-performance methods and cloud computing. In future research, we will also focus on non-coding genomic regions, we will continue to focus on rare variants in GWASs, and we will develop new methods to aid in the identification of the causes of complex diseases. With the rapid development of high-performance cloud computing techniques, these abilities should continue to improve.

## Figures and Tables

**Table 1:** The recall, precision, and F-measure of FDHE-IW using 12 disease loci with marginal effects (DME) models.

Models	100 SNPs (1600 Sample Size)			1000 SNPs (4000 Sample Size)		
	Recall	Precision	F-Measure	Recall	Precision	F-Measure
DME-1	26.09%	54.55%	35.29%	67.74%	84.00%	75.00%
DME-2	15.00%	27.27%	19.35%	75.00%	78.57%	76.74%
DME-3	44.83%	44.83%	44.83%	79.25%	55.26%	65.12%
DME-4	60.00%	33.96%	43.37%	92.21%	43.56%	59.17%
DME-5	69.62%	91.67%	79.14%	100.00%	85.22%	92.02%
DME-6	82.11%	78.00%	80.00%	100.00%	53.48%	69.69%
DME-7	96.00%	64.43%	77.11%	100.00%	35.46%	52.36%
DME-8	100.00%	31.35%	47.73%	100.00%	33.33%	50.00%
DME-9	95.00%	73.08%	82.61%	99.00%	73.88%	84.62%
DME-10	95.00%	93.14%	94.06%	99.00%	86.84%	92.52%
DME-11	98.41%	100.00%	99.20%	100.00%	100.00%	100.00%
DME-12	96.88%	97.89%	97.38%	98.85%	98.85%	98.85%

**Table 2:** Performance comparisons on *recall, precision*, and *F-measure* (100 SNPs, 1600 sample size). Runtimes are displayed as a mean value of models (multiplicative models: DME-1–DME-4, threshold models: DME-5–DME-8, and concrete models: DME-9–DME-12).

Models	Algorithms	Recall	Precision	F-Measure	Runtime(s)
Multiplicative model	FDHE-IW	36.5%	40.2%	35.7%	2.95
	MACOED	**68.5%**	**90.8%**	**71.7%**	10.8
	BEAM	14.8%	10.3%	7.7%	8.52
	BOOST	0.5%	62.5%	0.5%	**0.6**
	SNPHarvester	0.3%	50.0%	0.5%	2.97
Threshold model	FDHE-IW	86.9%	66.4%	71.0%	2.95
	MACOED	**98.0%**	**83.0%**	**89.3%**	11.40
	BEAM	84.5%	68.3%	60.5%	8.52
	BOOST	34.8%	99.0%	37.1%	**0.95**
	SNPHarvester	51.8%	47.0%	29.0%	2.92
Concrete model	FDHE-IW	96.3%	**91.0%**	**93.3%**	**2.95**
	MACOED	**98.8%**	**84.8%**	91.0%	11.59
	BEAM	81.3%	62.3%	70.1%	8.52
	BOOST	66.3%	87.3%	69.7%	**0.66**
	SNPHarvester	91.3%	57.3%	67.7%	2.90

FDHE-IW: A fast approach for detecting high-order epistasis with interaction weight; MACOED: Multi-objective ant colony optimization epistasis detection; BEAM: Bayesian epistasis association mapping; BOOST: Boolean operation-based screening and testing.

**Table 3:** Performance comparisons on *recall*, *precision* and *F-measure* (1000 SNPs, 4000 sample size). Runtimes are displayed as the mean value of models (multiplicative models: DME-1–DME-4, threshold models: DME-5–DME-8, and concrete models: DME-9–DME-12).

Models	Algorithms	Recall	Precision	F-Measure	Runtime(s)
Multiplicative model	FDHE-IW	**78.55%**	**65.35%**	**69.01%**	65.4
	MACOED	75.00%	32.85%	20.82%	440
	BEAM	35.12%	37.84%	17.65%	308
	BOOST	18.50%	22.95%	9.40%	4
	SNPHarvester	5.55%	32.20%	3.61%	130
Threshold model	FDHE-IW	**100.00%**	51.87%	**66.02%**	66.6
	MACOED	**100.00%**	14.31%	11.08%	450
	BEAM	98.46%	75.27%	42.64%	199
	BOOST	88.50%	62.50%	33.70%	9
	SNPHarvester	87.50%	29.45%	12.24%	143
Concrete model	FDHE-IW	**99.21%**	**89.89%**	**94.00%**	**35.3**
	MACOED	**100.00%**	38.60%	23.89%	303
	BEAM	66.94%	63.05%	31.68%	133
	BOOST	72.50%	20.83%	15.82%	6
	SNPHarvester	84.00%	**92.45%**	43.80%	62.3

**Table 4:** Three-way SNP combinations, SNP1, SNP2, and SNP3 were mapped to gene1, gene2, and gene3, respectively. (NA denotes that the corresponding SNP is situated within a non-coding region).

SNP1	Gene1	SNP2	Gene2	SNP3	Gene3	*G*-test *p*-Value
rs380390	*CFH*	rs1930022	NA	rs3913094	NA	2.22 × 10^−16^
rs380390	*CFH*	rs10504709	NA	rs2402053	NA	1.11 × 10^−16^
rs380390	*CFH*	rs10504709	NA	rs2380684	NA	1.11 × 10^−16^
rs380390	*CFH*	rs2380684	NA	rs2224762	*JMJD2C*	0
rs380390	*CFH*	rs10504548	NA	rs2224762	*JMJD2C*	0
rs380390	*CFH*	rs2402053	NA	rs2224762	*JMJD2C*	0
rs380390	*CFH*	rs718263	*NCALD*	*rs2224762*	*JMJD2C*	0
rs1329428	*CFH*	rs3775652	*INPP4B*	rs6598991	NA	2.22 × 10^−16^
rs725518	*RRM1*	rs3775652	*INPP4B*	rs1002979	NA	2.22 × 10^−16^

**Table 5:** *4-way* SNP combinations identified using a *G*-test.

snp1	Gen1	SNP2	Gen2	SNP3	Gen3	SNP4	Gen4	*G-*test *p*-Value
rs1740752	*PCCA*	rs4772270	NA	rs7044653	NA	rs6598991	NA	0
rs4772270	NA	rs7044653	NA	rs1329428	*CFH*	rs6598991	NA	0

**Table 6:** Comparison of the results of seven algorithms using the age-related macular degeneration (AMD) data set.

	FDHE-IW	BEAM	epi Forest	DCHE	FHSA-SED	NHSA-DHSC	epiACO
Relevant SNPs or genes identified	**SNPs:** rs380390 rs1329428 rs10511467 rs6598991 rs10507949 rs3776652 **Genes:** *CFH* *JMJD2C* *INPP4B*	**SNPs:** rs380390 rs1329428 **Gene:** *CFH*	**SNPs:** rs380390 rs1329428 rs1394608 rs7104698 **Gene:** *CFH*	**SNPs:** rs380390 rs1329428 rs1394608 rs1740752 rs1363688 rs10512174 rs618499 rs1926489 **Genes:** *CFH* *ZNF25* *SGCD* *LRIG3* *DRD1* *ISCA1*	**SNPs:** rs380390 rs1329428 rs10272438 rs1740752 rs3775652 rs1394608 rs1363688 rs10511467 **Genes:** *CFH* *BBS9* *SGCDINPP4B*	**SNPs:** rs380390 rs1329428 rs10272438 rs1363688 rs1394608 rs3775652 rs7104698 rs10511467 rs10512413 **Genes:** *CFH* *INPP4B* *BBS9* *ABL1ANKS1B*	**SNPs:** rs380390 rs1329428 rs1363688 rs1394608 rs2224762 rs9328536 rs943008 rs718263 **Genes:** *CFH* *MED27* *KDM4C* *NCALD* *NEDD9*

## Supplementary Materials

The following are available online at http://www.mdpi.com/2073-4425/9/9/435/s1, Supplementary File 1.xls: All the SNP combinations and gene combinations; Supplementary File 2.pdf: Table S1: Penetrance functions of the three DME epistasis models, Table S2: the parameters and the penetrance values of 12 DME models; Supplementary File 3.pdf: Figure S1: AUC curves of *4-way* SNP combination (SNP1: rs1740752 SNP2: rs4772270 SNP3: rs7044653 SNP4: rs6598991); Figure S2: AUC curves of *4-way* SNP combination (SNP1: rs4772270, SNP2: rs7044653, SNP3: rs1329428, SNP4: rs6598991); Supplementary Material 4: FDHE-IW.rar: The matlab source code of FDHE-IW algorithm.
