# Supplementary material for: FDHE-IW: A Fast Approach for Detecting High-Order Epistasis in Genome-Wide Case-Control Studies
Source: Genes (Basel). 2018 Aug 29;9(9):435. doi: 10.3390/genes9090435 (PMC6162554; doi:10.3390/genes9090435)
Supplement: Supplementary file 1 [file genes-09-00435-s001.zip › supplementary file1-3/Supplementary file 2.pdf]

FDHE-IW: a fast approach for detecting high-order epistasis in genome-wide case-control studies

Shouheng Tuo

School of Computer Science & Technology, Xi'an University of Posts & Telecommunications, 710121, China; tuo_sh@126.com

DME models

The DME (**disease loci with marginal effects**) model has both main effects and interaction effects. Twelve disease models (Model 1-Model 12) are composed of multiplicative model, threshold model and concrete model.

DME 1- DME 4 (H^2=0.005, MAF=0.05, 0.1, 0.2 and 0.5) are **multiplicative models** with two disease locus, in which the disease prevalence given the frequency of genotype combination increases multiplicatively with the incremental presence of the disease. The genetic heritability (H^2) of DME 1- DME 4 are all equal to 0.005, minor allele frequencies (MAF) of them equal 0.05, 0.1, 0.2 and 0.5, respectively.

DME 5- DME 8 (H^2=0.02, MAF=0.05, 0.1, 0.2 and 0.5) are the **threshold models** in which the prevalence of genotype frequency does not increase until the number of disease alleles pass the threshold).

DME 9- DME 12 (H^2=0.02, MAF=0.05, 0.1, 0.2 and 0.5) are **concrete model** used to mimic the effect that epistasis has on susceptibility to handedness and the color of swine (Marchini, et al., 2005; Neuman, et al., 1992).

Table S1 presents the **penetrance functions** of three models (**multiplicative models, threshold models, concrete model**) [1][2]

Table S1. Penetrance functions of the three DME epistasis models [2]

Model 1		Loci 1		
		AA	Aa	aa
Loci 2	BB	α	α	α
	Bb	α	$\alpha(1+\beta)^2$	$\alpha(1+\beta)^3$
	bb	α	$\alpha(1+\beta)^3$	$\alpha(1+\beta)^4$
Model 2		Loci 1		
		AA	Aa	aa
Loci 2	BB	α	α	α
	Bb	α	$\alpha(1+\beta)$	$\alpha(1+\beta)$
	bb	α	$\alpha(1+\beta)$	$\alpha(1+\beta)$
Model 3		Loci 1		
		AA	Aa	aa
Loci 2	BB	α	$\alpha(1+\beta)$	$\alpha(1+\beta)$
	Bb	$\alpha(1+\beta)$	α	α
	bb	$\alpha(1+\beta)$	α	α

Table S2. the parameters (H^2: genetic heritability, the disease prevalence P(D)) and the penetrance values of 12 DME models

DME	H^2	MAF	P(D)	AABB	AABb	AAbb	AaBB	AaBb	Aabb	aaBB	aaBb	aabb
DME -1	0.005	0.05	0.1	0.098	0.098	0.098	0.098	0.299	0.522	0.098	0.522	0.912
DME -2	0.005	0.1	0.1	0.096	0.096	0.096	0.096	0.197	0.282	0.096	0.282	0.405
DME -3	0.005	0.2	0.1	0.092	0.092	0.092	0.092	0.144	0.181	0.092	0.181	0.227
DME -4	0.005	0.5	0.1	0.078	0.078	0.078	0.078	0.105	0.122	0.078	0.122	0.142
DME -5	0.02	0.05	0.1	0.096	0.096	0.096	0.096	0.533	0.533	0.096	0.533	0.533
DME -6	0.02	0.1	0.1	0.092	0.092	0.092	0.092	0.319	0.319	0.092	0.319	0.319
DME -7	0.02	0.2	0.1	0.084	0.084	0.084	0.084	0.21	0.21	0.084	0.21	0.21
DME -8	0.02	0.5	0.1	0.052	0.052	0.052	0.052	0.137	0.137	0.052	0.137	0.137
DME -9	0.02	0.05	0.1	0.08	0.192	0.192	0.192	0.08	0.08	0.192	0.08	0.08
DME -10	0.02	0.1	0.1	0.072	0.164	0.164	0.164	0.072	0.072	0.164	0.072	0.072
DME -11	0.02	0.2	0.1	0.061	0.146	0.146	0.146	0.061	0.061	0.146	0.061	0.061
DME -12	0.02	0.5	0.1	0.067	0.155	0.155	0.155	0.067	0.067	0.155	0.067	0.067

Reference

[1] Marchini, J., et al. (2005) Genome-wide strategies for detecting multiple loci that influence complex diseases, Nature genetics, 37, 413-417.

[2] Jing P J, Shen H B. MACOED: a multi-objective ant colony optimization algorithm for SNP epistasis detection in genome-wide association studies[J]. Bioinformatics, 2015, 31(5):634-641.
