# Supplementary material for: FDHE-IW: A Fast Approach for Detecting High-Order Epistasis in Genome-Wide Case-Control Studies
Source: Genes (Basel). 2018 Aug 29;9(9):435. doi: 10.3390/genes9090435 (PMC6162554; doi:10.3390/genes9090435)
Supplement: Supplementary file 1 [file genes-09-00435-s001.zip › supplementary file1-3/Supplementary file 3.pdf]

FDHE-IW: a fast approach for detecting high-order epistasis in genome-wide case-control studies

Table 5. *4-way* SNP combinations identified using a G-test.

snp1	Gen1	snp2	Gen2	snp3	Gen3	snp4	Gen4	*p*-value
rs1740752	PCCA	rs4772270	NA	rs7044653	NA	rs6598991	NA	0
rs4772270	NA	rs7044653	NA	rs1329428	CFH	rs6598991	NA	0

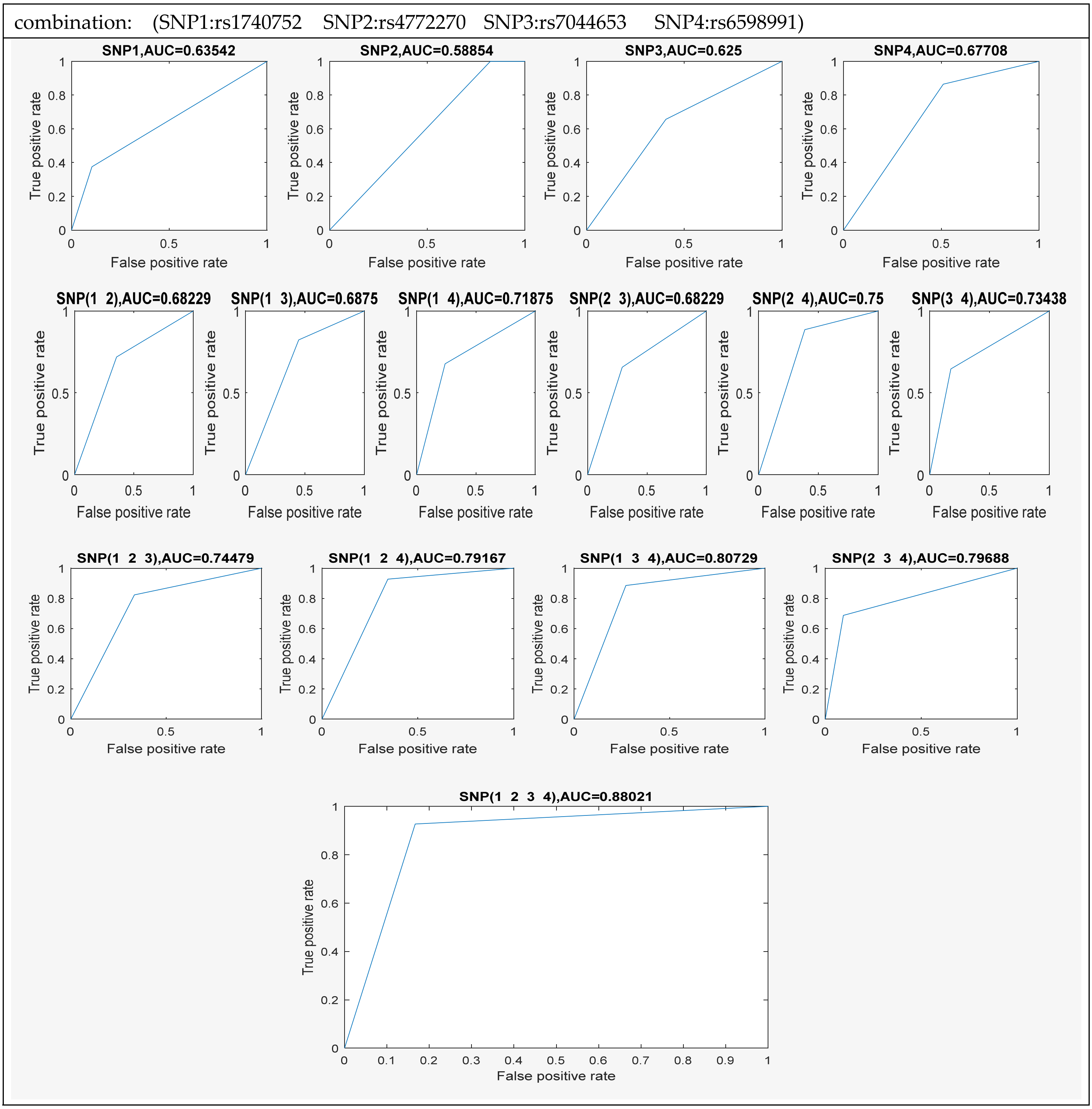

Figure S1. AUC curves of 4-way SNP combination
(SNP1: rs1740752 SNP2: rs4772270 SNP3: rs7044653 SNP4: rs6598991)

SNP combination (SNP1:rs4772270, SNP2: rs7044653, SNP3:rs1329428, SNP4:rs6598991)

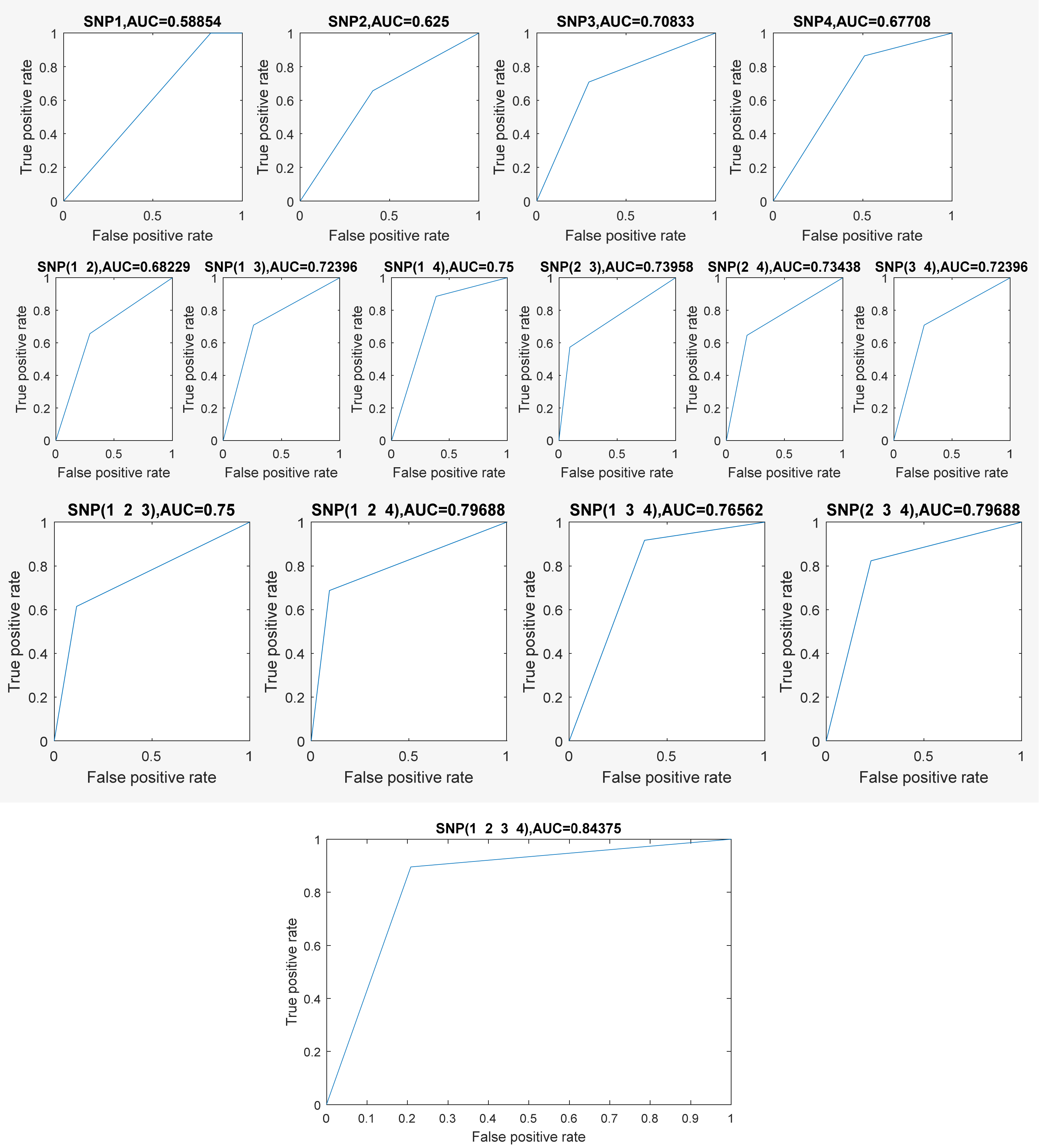

Figure S2. AUC curves of 4-way SNP combination

(SNP1: rs4772270, SNP2: rs7044653, SNP3: rs1329428, SNP4: rs6598991)
